# Supplementary material for: MetaRibo-Seq measures translation in microbiomes
Source: Nat Commun. 2020 Jun 29;11:3268. doi: 10.1038/s41467-020-17081-z (PMC7324362; doi:10.1038/s41467-020-17081-z)
Supplement: Supplementary file 10 — Supplementary Data 7 [file 41467_2020_17081_MOESM10_ESM.zip › File2/Confidence_High_Taxonomy/417387_out.krona.html]

Javascript must be enabled to view this page.

members
magnitude
magnitudeUnassigned
count
unassigned
taxon
rank

417387\_out

16

16
2
superkingdom

phylum
16
976

16
200643
class

171549
16
order

family
1
815

816
1
genus

1
821
species

SRS024492\_contig\_number\_15013

family
1853231
15

genus
283168
15


SRS147139\_contig\_number\_12960
species
626933
1

species

SRS018888\_contig\_number\_contig-100\_1249.57634SRS019161\_contig\_number\_24410SRS019397\_contig\_number\_contig-100\_1130.84516SRS019445\_contig\_number\_33939SRS019445\_contig\_number\_contig-100\_693.179021SRS020328\_contig\_number\_37343SRS024492\_contig\_number\_contig-100\_1450.106139SRS044535\_contig\_number\_24971SRS076929\_contig\_number\_contig-100\_1158.145157SRS077730\_contig\_number\_18161SRS1041118\_contig\_number\_10872SRS143876\_contig\_number\_15425SRS147557\_contig\_number\_30619SRS149181\_contig\_number\_29064
28118
14
